# The Influence of Competition Day Loads on the Metabolic and Immune Response of Olympic Female Beach Volleyball Athletes: A Sportomics Analysis

**DOI:** 10.3390/nu17111924

**Published:** 2025-06-04

**Authors:** Renan Muniz-Santos, Flavio Bachini, Adriana Bassini, P. C. B. Alexandre, Igor Jurisica, Vinod Chandran, L. C. Cameron

**Affiliations:** 1 Lorraine Protein Biochemistry Group, Graduate Program in Neurology, Gaffrée e Guinle University Hospital, Rio de Janeiro 20270-004, RJ, Brazil; renanmuniz@edu.unirio.br (R.M.-S.); pedro.alexandre@unirio.br (P.C.B.A.); 2 Laboratory of Protein Biochemistry, The Federal University of the State of Rio de Janeiro, Rio de Janeiro 20290-250, RJ, Brazil; f.bachini@gmail.com (F.B.); bassiniadriana@gmail.com (A.B.); 3 Osteoarthritis Research Program, Division of Orthopedic Surgery, Schroeder Arthritis Institute and Data Science Discovery Centre for Chronic Diseases, Krembil Research Institute, University Health Network, Toronto, ON M5T 0S8, Canada; juris@ai.utoronto.ca; 4 Departments of Medical Biophysics and Computer Science, Faculty of Dentistry, University of Toronto, Toronto, ON M5G IL7, Canada; 5 Institute of Neuroimmunology, Slovak Academy of Sciences, 845 10 Bratislava, Slovakia; 6 Gladman-Krembil Psoriatic Arthritis Program, Schroeder Arthritis Institute, Krembil Research Institute, University Health Network, Toronto, ON M5T 0S8, Canada; vinod.chandran@uhn.ca; 7 Division of Rheumatology, Department of Medicine, Institute of Medical Science, Department of Laboratory Medicine and Pathobiology, Faculty of Medicine, University of Toronto, Toronto, ON M5T 0S8, Canada

**Keywords:** female athlete, acute phase response, amino acid, sportomics, central fatigue, beach sports, exercise metabolism, immune system

## Abstract

**Background:** Beach volleyball (BVb) is a highly demanding Olympic sport characterized by intense physical activity and unique environmental challenges, including varying weather conditions and sandy, unstable court surfaces. Despite its popularity, there is a notable lack of scientific research addressing the metabolic and immune responses of elite female athletes in this sport. This study aims to address this gap by investigating two world-class Olympic medalists, female BVb players, who represent a country with a rich history in the sport. **Methods:** Two athletes underwent a simulated competition day consisting of two matches. A standardized protocol was utilized to collect blood and urine samples at seven time points, allowing for analysis throughout the competition and recovery phases. The analysis included various electrolytes, as well as hematological, metabolic, and inflammatory markers. Additionally, we assessed selected hormones, such as insulin, serotonin, ACTH, and cortisol, along with amino acids related to energy metabolism and neurotransmitter synthesis. **Results:** Both athletes presented a trend toward electrolyte disturbances, especially hypokalemia, with a mean decrease of 15% and individual values reaching as low as 3.3 mmol/L post-match. This indicates that BVb may pose a risk for such disturbances. Additionally, the matches led to 20% to 60% increases in muscle injury markers, with incomplete recovery even after a day of rest, signaling persistent physiological stress post-competition. This increase was matched by stimulating stress hormones (ACTH and cortisol rose up to 4-fold and 3-fold, respectively), and markers of exercise intensity, such as lactate and ammonium. Moreover, the simulated BVb competition day impacted the amino acid response, with the Fischer ratio (BCAA/AAA) and blood tryptophan decreasing to a minimum of 60% of the initial levels and blood serotonin increasing by up to 180%, which are signs of an increased risk of central fatigue onset, according to the Fischer and Newsholme theory. **Conclusions:** The responses examined in this exploratory study contribute to a deeper understanding of the metabolic and immune demands placed on elite female BVb players, suggesting practical applications. By addressing the similar physiological responses observed among the athletes and emphasizing their unique individual responses—despite following the same protocol under identical conditions and sharing similar life habits for an extended period—this study highlights the critical necessity for the n-of-1 monitoring of athletes.

## 1. Introduction

Similar to volleyball, beach volleyball (BVb) is an Olympic sport characterized by multiple bouts of intense movement followed by intermittent periods of rest. However, the beach environment presents additional challenges, such as an unstable, sandy court surface, which can significantly affect the exercise load [1]. Furthermore, the outdoor nature of beach volleyball exposes athletes to various weather conditions, including a wide range of temperatures, humidity, wind, and solar radiation—factors that can notably influence their metabolic demands [2,3]. For instance, a recent study evaluating elite athletes of different sports—including BVb—showed that heat and humidity conditions (31.9 ± 1.6 °C; relative humidity, 72% ± 5%) significantly decrease the time to exhaustion [4].

Despite a growing body of scientific research, there is a notable lack of published studies evaluating the metabolic and immune demands of BVb and other beach sports, with orthopedic and dermatological injuries remaining the primary focus [5,6]. This gap is even more pronounced in elite athletes due to a lack of incentives to publish data and competitive secrets [7]. Moreover, women are often under-represented in studies of athletic performance and exercise-induced metabolic stress responses, similarly to other research studies. In this context, there is a clear need for investigations into the exercise-induced metabolic response in female high-level beach sport athletes, as translating data from comparable indoor sports or male athletes can be imprecise.

Indeed, examining the immunometabolic response to exercise necessitates multiple perspectives. First, the type of sport significantly influences its metabolic demands, shaped by factors such as activity duration, the frequency and intensity of sprints and jumps, and sport-specific movements. Second, external conditions—including temperature, terrain, humidity, sun exposure, and wind—have a substantial impact on these responses. Lastly, each athlete also presents unique conditions and responses based on individual characteristics, which may include their anthropometrics, genetics, training level, and psychological factors [7]. Therefore, to fully understand an athlete’s response, it is essential to analyze the effects of specific exercise protocols or sports while acknowledging the individual differences among athletes, also known as the external and internal loads [8].

To assess the exercise load and the metabolic response elicited by stressors, sportomics approaches have been implemented to inform hands-on decision-making for athletes and advance scientific research [9,10]. In these investigations, we prioritize ex post facto analysis in the field of play within the uncontrolled environments that athletes face, rather than relying on mechanistic controlled studies. This is particularly important in BVb, given the high exposure to uncontrolled environmental conditions. While this in-field approach presents inherent limitations in establishing causality, it has proven to be an effective way to monitor athletes, describe their responses, generate different hypotheses for testing, and support decision-making processes [11,12]. For additional reviews on sportomics, please refer to Sellami et al. (2021) and Bragazzi et al. (2020) [8,12].

In this context, we aim to conduct a sportomics investigation into the metabolic and immune responses during various phases of a beach volleyball competition day among Olympic female medalists. Additionally, we will compare these responses to those observed in other exercise models, particularly within beach sports, when possible. Given that we hypothesize that the athletes may display differing responses in selected analytes even under the same protocol, we will explore these potential differences to highlight the significance of evaluating internal loads and conducting n-of-1 investigations in exercise studies.

## 2. Materials and Methods

### 2.1. Participants

Two world-class female beach volleyball players (aged 33, with heights of 1.74 cm and 1.81 cm, body weights of 69.0 kg and 65.0 kg, respectively, and 12 years of professional experience) were evaluated during a simulated competition day, conducted one year before the Rio 2016 Summer Olympic Games.

This study followed the guidelines for research involving human subjects (Health National Council, Brazil, 1996). The Ethics Committee of Human Research at the Federal University of the State of Rio de Janeiro (Rio de Janeiro, Brazil) approved the trial (protocol 117/2007, renewed in 2011, 2013, and 2016). Both athletes were fully informed about all the procedures and provided written consent before participating in the experiment.

### 2.2. Experimental Design

**Figure 1:**
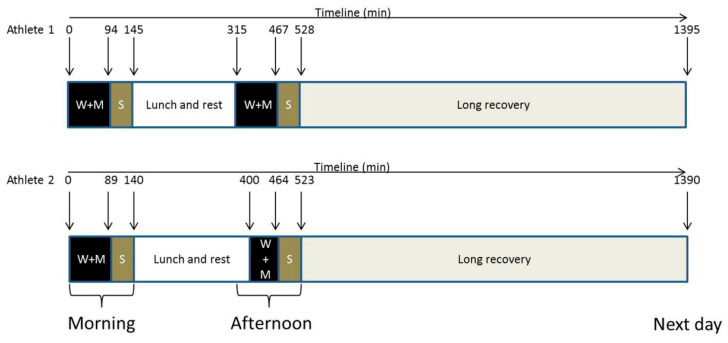
Experimental design. The athletes played two matches with short recoveries and a long recovery following their usual routine for a day of competition. It was noted that during the afternoon, Athlete 1 started the bout 45 min before Athlete 2. W + M = warming and match; S = short recovery; downward arrow = blood and urinary collection.

The study assessed the impact of playing two matches on the same day, followed by a long recovery period the next morning. The athletes were instructed to maintain their usual nutrition and sleep routines, as they would on a typical competition day. Each match was preceded by a 15 min warm-up, followed by a two-set match in the morning and a three-set match in the afternoon. The athletes provided seven blood and urine samples: pre-match morning (09:00; UTC-3); post-match morning; 60 min after the morning match (short recovery 1; SRec1); pre-match afternoon; post-match afternoon; 60 min after the afternoon match (short recovery 2; SRec2); and with a final long recovery of roughly 15 h (long recovery) (Figure 1). Regarding the sampling time points, they were not selected based on the kinetics of specific markers but rather to assess the metabolic response at key moments for the athlete (such as pre-match 1 and 2, post-match 1 and 2). Short recovery time points were chosen to be approximately consistent with other sportomics analyses. The long-term recovery sample was collected for convenience during the athletes’ visit to our session.

One month before the simulated competition day, both athletes were assessed for their hematological parameters, injury biomarkers, and nutritional status to ensure homogeneity. We evaluated the total protein and albumin, cholesterol, LDL, HDL, triacylglycerol, iron, ferritin, transferrin, total iron-binding capacity, red and white blood cells, ammonium, urea, urate, creatinine, sodium, potassium, chloride, phosphorus, magnesium, calcium, alanine aminotransferase (ALT), alkaline phosphatase (ALP), aspartate aminotransferase (AST), total creatine phosphokinase (CK), lactate dehydrogenase (LDH), gamma-glutamyltransferase (GGT), creatine phosphokinase-MB (CK-MB), C-reactive protein, alpha-1 glycoprotein, and haptoglobin. The levels of analytes were similar between the athletes.

The athletes were asked about their menstrual cycle phase and contraceptive use. Athlete 1 reported that she had completed her menstrual cycle 17 days before this evaluation and did not use any contraceptives. Meanwhile, Athlete 2 had completed her cycle 24 days before the trial but was using a Mirena^®^ IUD (Bayer, Leverkusen, Germany), which contains 52 mg of levonorgestrel with a release rate of 20 µg/24 h.

### 2.3. Blood Sampling and Urinary Collection

Venipunctures were drawn from the antecubital vein into Vacuette tubes (Greiner Bio-One, Frickenhausen, Germany) and centrifuged at 3000× *g* for 10 min (RDE i model B-40). The urine, serum, and plasma samples were aliquoted into cryotubes, stored at −80 °C, and analyzed by a commercial laboratory.

Biochemical analyses were conducted using the following methods: ALT, ALP, AST, CK, LDH, GGT, glucose, lactate, ammonium, urate, and urea were assessed by enzymatic methods; myoglobin (MB) and CK-MB by enzyme immunoassay; creatinine by colorimetric methods; C-reactive protein by the turbidimetric method; alpha-1 glycoprotein (A1AG) by nephelometry; chloride and magnesium by colorimetric methods; sodium and potassium by the ion-selective electrode method; prolactin, insulin, cortisol, and adrenocorticotropic hormone (ACTH) by chemiluminescence; serotonin and amino acids by high-performance liquid chromatography (HPLC); and urine by flow cytometry and microscopy (Table 1).

### 2.4. Diet and Supplementation

The athletes were supplemented for 10 months prior to the trial with 100% Whey Protein Isolate, taking one scoop mixed with approximately 200 to 300 mL of water per day after their afternoon training session (Dymatize^®^, Dallas, TX, USA). Additionally, before each morning and afternoon training session, they consumed three pills of BCAA—Complex 2200 (Dymatize^®^, Dallas, TX, USA). β-alanine was taken as one pill before lunch. These supplements were identical for both athletes; however, they were also advised by their technical team to take different supplement batches.

Athlete 1 was instructed by her coach to consume one scoop of Endurox R4^®^ mixed with approximately 500 mL of water per day during all training sessions (Pacific Health^®^, Woodbury, NJ, USA) and 30 g of Carb Up™ per training session (Probiótica, São Paulo, Brazil). Athlete 2 consumed one scoop of lemon-flavored Accelerade^®^ mixed with 500 mL of water per day (Pacific Health^®^, Woodbury, NJ, USA). All these supplements were incorporated into the athletes’ regular daily diet, as described below.

Both athletes were advised to eat and drink as they would on a competition day, following the nutritional guidance provided by their technical team. On the competition day, Athlete 1’s food consumption was estimated to provide an equivalent of ~10,200 kJ, (31% derived from protein, 55% from carbohydrates, and 14% from fat) while hydrating with 3.0 L of water. Athlete 2 consumed an equivalent of ~11,200 kJ (29.5% derived from protein, 49% from carbohydrates, and 21.5% from fat) along with 2.5 L of water throughout the day. The energy and macronutrient distributions were calculated using a national Brazilian table, which considers the average food amount.

### 2.5. Data Analysis

Given the sample size and the in-field, ex post facto nature of the study, no inferential statistics were performed, characterizing this as an exploratory study. The data were normalized relative to the pre-match 1 time point and reported as percentage changes, as previously described in detail [13].

## 3. Results

### 3.1. Red Sector Parameters

**Figure 2:**
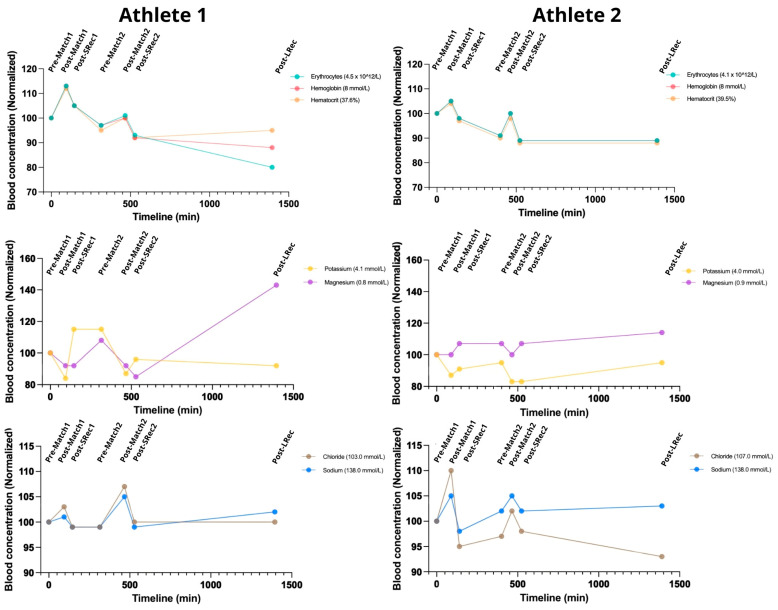
The simulated day of competition had a comparable impact on the athletes concerning red sector parameters and electrolytes. The data from each athlete at all the time points are normalized to the pre-match 1 values. The reference range for a normal population: hemoglobin 7.4–9.9 mmol/L; hematocrit: 36–48%; potassium: 3.5–5.0 mmol/L; magnesium: 0.8–1.1 mmol/L; sodium 135–145 mmol/L; chloride 96–106 mmol/L.

Red sector parameters were measured to assess acute blood volume changes (Figure 2) indirectly. The matches induced acute raises in hemoglobin (Hb) and hematocrit (Ht), which returned toward the baseline during short recovery 1 (SRec1) and short recovery 2 (SRec2). Both athletes maintained Hb and Ht levels predominantly below the initial values throughout the protocol, except during post-match 1. Creatininemia also increased after the matches, followed by a recovery trend, with the increases ranging from 17 to 35% (Figure 2). As expected, the changes in urine specific gravity (USG) were shifted to the right (Table 2).

The matches led to increased sodium and chloride blood concentrations of up to 5% and 10%, respectively (Figure 2). Natremia hit the upper limit of the standard values (145 mmol/L) for both athletes following post-match 2. Meanwhile, kalemia dropped during the protocol to values below the reference range for both athletes (a minimum of 3.4 mmol/L for Athlete 1 and 3.3 mmol/L for Athlete 2), showing a subsequent recovery trend. For Athlete 1, magnesium decreased during the matches, while it remained stable for Athlete 2. At long recovery (LRec), magnesium increased ~70% in Athlete 1 and 10% in Athlete 2.

### 3.2. Muscle and Liver Injury Markers

**Figure 3:**
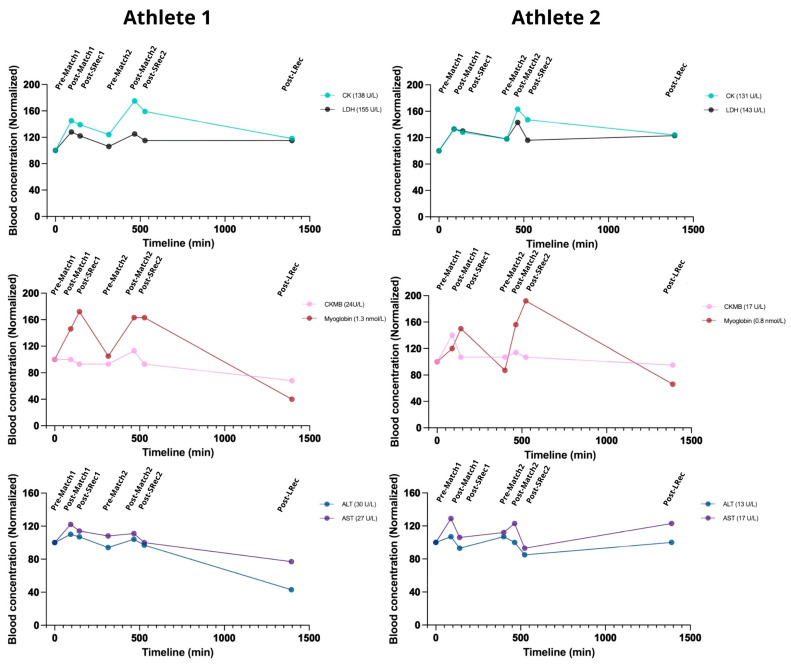
The games acutely affected cell damage biomarkers, with incomplete recovery observed during the analyzed recovery period. ALT: alanine aminotransferase; ALP: alkaline phosphatase; AST: aspartate aminotransferase; CK: total creatine phosphokinase; LDH: lactate dehydrogenase; GGT: gamma-glutamyltransferase, CKMB: creatine phosphokinase–MB. The reference range for a normal population: CK 30–135 U/L; LDH 100–200 U/L; MB 1.6–3.7 nmol/L; CKMB 5–25 U/L; AST 10–30 U/L; ALT 10–40 U/L; ALP 30–120 U/L; GGT 2–30 U/L; potassium: 3.5–5.0 mmol/L; magnesium: 0.8–1.1 mmol/L; sodium 135–145 mmol/L; chloride 96–106 mmol/L.

Muscle microinjury markers are valuable in managing exercise loads. After the first match, both athletes experienced significant increases from 20 to 45% in CK, LDH, Mb, and CKMB levels, with incomplete recovery during the subsequent short and long recovery sessions (SRec1, SRec2, and LRec) (Figure 3). Myoglobin exhibited additional increases during SRec1 and SRec2. However, CKMB and Mb had recovered to their initial levels before the second match, unlike CK and LDH.

Similarly to the first match, the second match promoted an increase in all the markers (ranging from 35 to 60% for CK and Mb and ~20% for LDH and CKMB). Myoglobin fully returned to pre-match levels during LRec, while the CK and LDH levels were still ~20% higher than pre-match levels.

AST and ALT exhibited similar kinetics, increasing acutely during exercise, with a recovery trend during SRec1 and SRec2 (Figure 3). Similarly, GGT and ALP, two controls for hepatic and biliary health, followed similar kinetics. During LRec, Athlete 1 showed a more pronounced decrease in the evaluated markers than Athlete 2. However, it is essential to note that the absolute initial values of Athlete 1 were higher than those of Athlete 2.

### 3.3. Inflammatory Response

**Figure 4:**
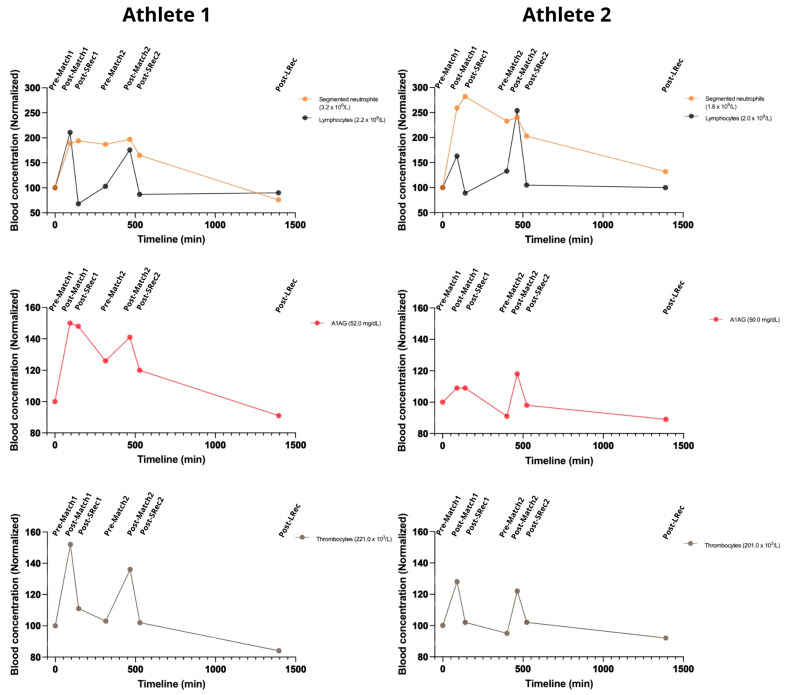
The games elicited an increase in white blood cell counts, thrombocytes, and A1AG, with variable magnitudes, demonstrating a consistent acute phase response. A1AG: alpha-1 acid glycoprotein. The reference range for a normal population: neutrophils 1.5–8.0 × 10^9^ per liter; lymphocytes 1.0–4.8 × 10^9^ per liter; A1AG 50 to 120 mg/dL; thrombocytes 150–450 × 10^9^ per liter.

The first match led to an increase in WBC for both athletes. The athletes had similar responses to the matches but with different ratios. Neutrophils raised (up to 200–250%) during match 1. They remained high until the end of match 2, slowly decreasing during Lrec (Figure 4). Lymphocyte counts reflected a more acute response to exercise, increasing in both athletes, with a quick drop to pre-protocol levels during SRec. Also, both A1AG and thrombocytes increased after each match, though the magnitude of this increase differed among the athletes. Athlete 1 exhibited higher peaks for both markers after the first match, with increases of 50% for A1AG and 52% for thrombocytes, compared to 9% and 28% in the other athletes. Following the second match, both analytes rose again, reinforcing the presence of a consistent acute phase response to competitive play (Figure 4).

### 3.4. Hormonal Response

**Figure 5:**
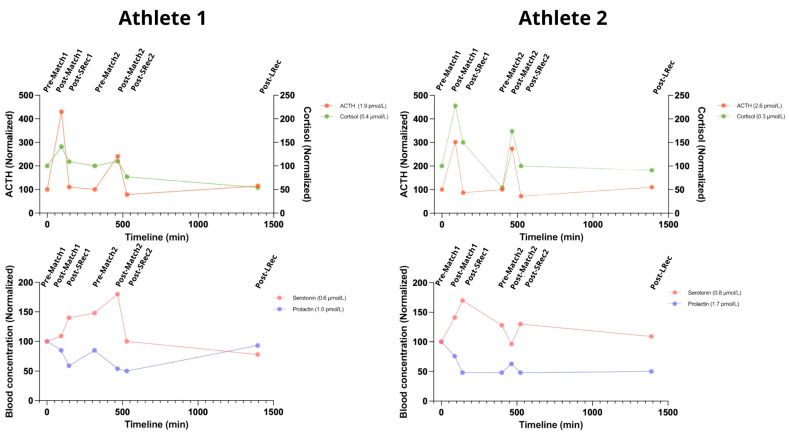
The considerable rise in hormones associated with the stress response, alongside the onset of central fatigue during exercise, underscores the systemic physiological impact of the protocol. ACTH: adrenocorticotropic hormone. The reference range for a normal population: ACTH 2 to 11 pmol/L; cortisol 0.140 to 0.690 μmol/L; serotonin 0.28–1.14 µmol/L; prolactin 3–25 ng/mL.

ACTH levels rose dramatically after both matches, increasing by 330% for Athlete 1 and 200% for Athlete 2 following the first match and by 135% and 175%, respectively, after the second match. Cortisolemia mirrored this pattern, although with varying magnitudes. After the first match, cortisol increased by 40% in Athlete 1 and 130% in Athlete 2 (reaching the standard upper limit of 0.690 μmol/L). The second match produced even higher increases, with cortisol levels climbing by 165% for Athlete 1 and 222% for Athlete 2. The short recovery sessions were sufficient to promote significant reductions in ACTH and cortisol levels (Figure 5).

Both athletes exhibited notable increases in blood serotonin following intense competition, with Athlete 1 peaking at 180% after the second match and Athlete 2 reaching 170% during Srec1 (with levels at 1.36 µmol/L, exceeding the normal upper limit). Exceptionally, Athlete 2 exhibited an immediate decrease after the second match, followed by an increase during SRec2, potentially indicating a delayed response. Both athletes experienced significant declines during the long recovery phase, dropping to 78% for Athlete 1 and 109% for Athlete 2.

### 3.5. Nitrogenous Compounds and Carbohydrates

**Figure 6:**
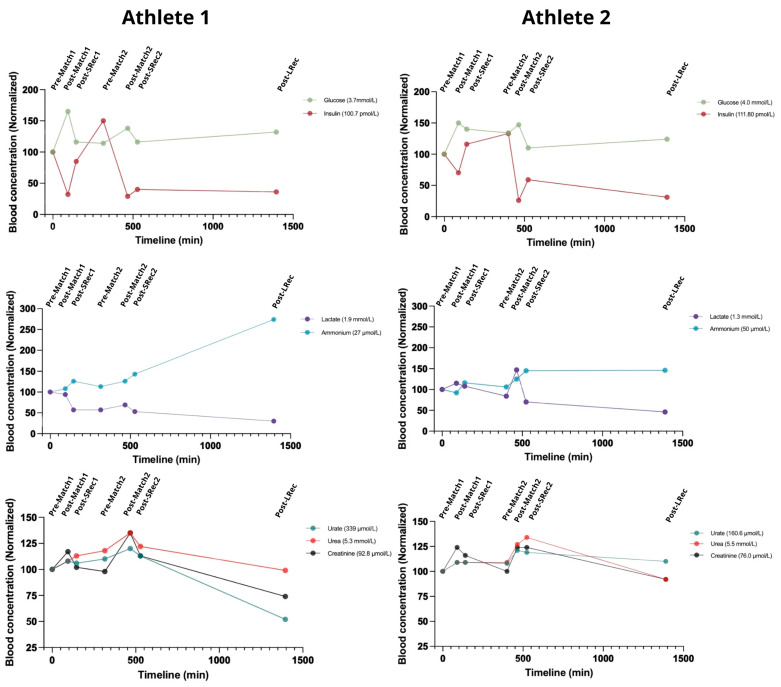
Classical metabolic markers highlight the high-intensity nature of BVb. The reference range for a normal population: glucose 3.9–5.6 mmol/L; insulin 30–90 pmol/L; lactate 0.5 to 2.2 mmol/L; ammonium 11–47 µmol/L; urate 155–357 µmol/L; urea 2.8–7.2 mmol/L; creatinine 44–97 μmol/L.

The gluco-insulinic response was similar among the athletes. Insulin dropped after both matches, reaching approximately 30% of initial levels after the second match for both athletes (Figure 6). Concurrently, glucose increased 35–50% above initial concentrations during the matches. The matches also led to a considerable increase in lactate, with the second match eliciting a greater response, particularly in Athlete 2 (75% compared with 21% in Athlete 1). Conversely, the first match did not impact Athlete 1’s lactatemia considerably (Figure 6).

Blood ammonium (for clarity in this paper, we will denote the combined concentrations of ammonium and ammonia as ammonium) is a key marker of exercise-induced metabolic stress. The matches led to accumulative increases in blood ammonium, which continued to rise during recovery. Athlete 1 experienced a significant spike during LRec, ending the protocol with an ammonium level of 275% compared to the initial concentration, while Athlete 2 had a 1.5-fold concentration. At LRec, both athletes exhibited ammonium concentrations roughly 50% above the upper normal limit, reaching about 75 mmol/L. Urate levels exhibited less fluctuation, increasing by approximately 10% for both athletes during the matches. The urate recovery rate differed, with Athlete 2’s levels remaining 10% higher than the initial concentration, while Athlete 1 finished 50% lower. The athletes presented similar blood urea responses, with acute increases during exercise (10–20%) (Figure 6).

### 3.6. Amino Acid Metabolism

**Figure 7:**
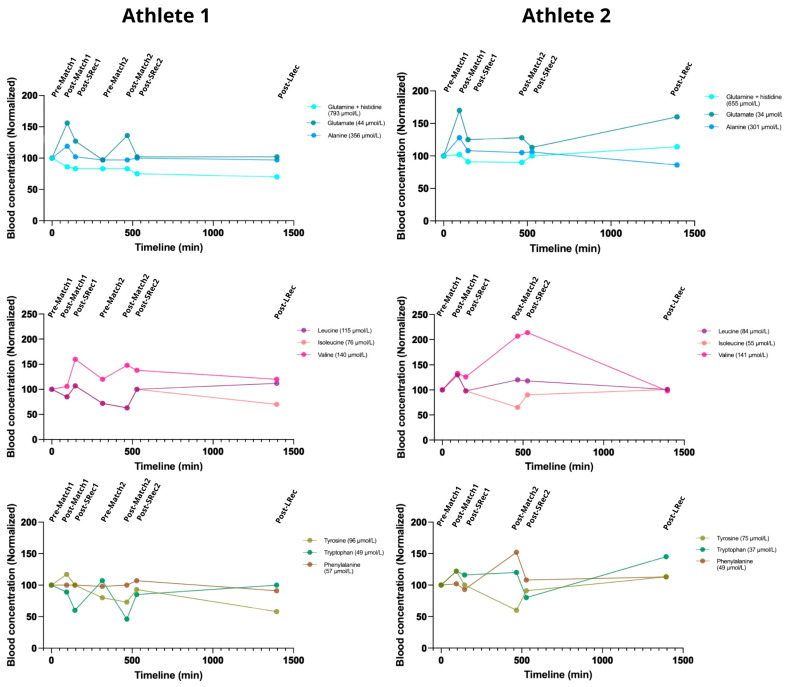
The amino acid responses related to energy metabolism, neurotransmitter synthesis, and ammonium transport were significantly affected by a simulated day of competition in BVb.

Most of the measured blood concentrations of amino acids were lower in Athlete 2. Alanine, glutamate, and glutamine are essential amino acids for energy production. The alanine concentration increased acutely after the first match for both athletes (18% and 29%, respectively) but remained unchanged after the second match. Glutamate showed the largest exercise-induced increase following the first match (56% and 70% for Athletes 1 and 2) with a rapid recovery. After the second match, glutamate increased by 42% for Athlete 1. Glutamine steadily decreased in Athlete 1 throughout the protocol, reaching 70% of the initial concentration by the LRec phase, while it remained relatively stable in Athlete 2 (Figure 7).

Athlete 1 exhibited a decrease in both leucine and isoleucine blood concentrations, returning to basal values after the SRec1. Those amino acids dropped to 70% of the initial concentration before the second match. The concentrations of these amino acids exhibited a mirroring effect during the first match for Athlete 2. Interestingly, both athletes responded similarly to leucine and isoleucine metabolism during the first match, while they were divergent in the second match. Valine metabolism was more affected by exercise in Athlete 2 than in Athlete 1.

In Athlete 1, the aromatic amino acid phenylalanine remained unchanged during the protocol, while Athlete 2’s phenylalanine concentration rose ~60% during the second match (simultaneously with a ~40% drop in tyrosinemia). Exercise caused an increasing drop in tryptophan concentration in Athlete 1, while match 1 caused a 25% increase in tryptophan in Athlete 2.

### 3.7. Selected Divergent Responses Among the Athletes

**Figure 8:**
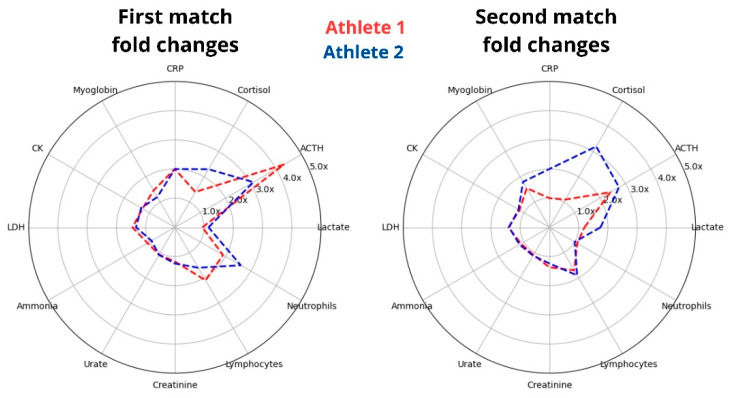
The athletes presented different acute responses to the same exercise protocol. The radar graph illustrates the fold changes in selected biomarkers from pre- to post-matches, with the data presented on the left for the first match and the right for the second match.

To assess athlete’s differences in internal load, it is essential to evaluate athletes under the same or similar external conditions. Our study standardized the protocol and analyzed two World Champion athletes who have trained together for many years, sharing their living environment, routines, and eating habits. Despite these similarities, we found varied acute responses in selected biomarkers, particularly in magnitude (Figure 8). This suggests that, even though they share similar conditions, there are differences in their internal loads in response to the same training protocol.

## 4. Discussion

The analyzed protocol involved a simulated day of beach volleyball competition with two world-class female athletes.

### 4.1. Red Sector Parameters and Electrolytes

Hematocrit increased acutely following the matches and returned to pre-exercise values quickly within the short recovery sessions, showing similar patterns for both athletes. In our study, the athletes exhibited similar hematocrit changes, and the observed differences regarding their metabolic and immune responses cannot be attributed to hemoconcentration or dilution. Hematocrit and selected major blood proteins are commonly used to assess acute changes in volemia during exercise and anti-doping controls [14]. Creatinine is a widely used marker of renal perfusion during exercise, which is also influenced by volemia [10]. Creatinine exhibited the same kinetics as hematocrit in both athletes, which further substantiates the notion that volemia changes did not influence the observed measured differences here.

Volemia and electrolyte changes are often linked, although changes in electrolyte concentrations can occur independently. While even minor acute sodium changes can be harmful due to its narrow normal range, we did not observe sodium changes exceeding 5% in our protocol. In our study, potassium and magnesium were the most impacted electrolytes. Remarkably, the athletes presented a consistent trend toward transient hypokalemia after the matches (from ~4.0 mmol/L to ~3.4 mmol/L on average). Evaluating electrolyte changes is crucial to understanding sports since beach sports players are known to be at higher risk for these disturbances due to exposure to sun radiation, high temperatures, and humidity [15]. We previously measured significant differences in electrolyte concentrations by comparing the effects of sunscreen or UV-protective long-sleeve shirts on Olympic Gold Medal beach volleyball players. Also, we previously found a similar hypokalemia trend with minor changes in chloride and sodium in a Windsurfing champion, who experienced weather conditions similar to BVb players [16]. This transient hypokalemia has often been found in different sportomics studies in various sports, and its connection to environmental conditions is being increasingly explored [16]. In fact, kalemia if influenced by factors such as sweat rates and insulin and catecholamine release affecting blood-cell potassium shifts, as well as aldosterone and anti-diuretic hormone volemia regulation impacting urine and potassium excretion. All these underlying physiological and biochemical effects have an intense interplay with environmental conditions, which can influence exercise-induced hormone secretion, metabolic stress, and changes in the blood volume [7].

### 4.2. Cell Damage Markers

Both BVb matches increased markers of myocyte damage (CK, LDH, MB, CKMB), highlighting the sport’s potential for inducing muscle microinjury [17,18], with concurrent increases in AST, ALT, GGT, and ALP of up to 50% in the post-exercise samples. While MB was the most affected marker, it decreased quickly, returning to pre-protocol levels before the second match and LRec. The fluctuations suggest that the stress response to a BVb competition day can be systemic, indicating both myocyte and hepatocyte damage, as we previously demonstrated in soccer [19,20]. Therefore, recovery time is also essential in BVb when managing exercise load and training. In this context, we found that the short recovery sessions (SRec1 and SRec2) proved inadequate for the athletes’ full recovery, as evidenced by the incomplete normalization of CK and LDH and additional increases in MB, suggesting ongoing muscle stress. Consequently, the second match increased these markers to concentrations above those identified at post-match 1, demonstrating a cumulative strain. Similarly to our finding, MB was already found to increase, even up to ~240%, after a soccer match and has been associated with acceleration and deceleration [18,21]. It is important to note that exercise-induced muscle microinjury is an effect that, in beach sports, can be increased by several short displacements and eccentric loads on uneven, sandy surfaces [1]. CK and LDH remained elevated by ~20% at the end of the protocol, contrasting the MB value’s fast return to basal. This incomplete recovery of muscle markers at both the SRecs and the LRec is crucial since their levels are often associated with low exercise tolerance, pre-clinical injury, and overtraining syndrome [22,23].

### 4.3. Inflammatory and Hormonal Response

We measured inflammatory markers, including cells and fragments (neutrophils, lymphocytes, and thrombocytes) and protein (A1AG), recognizing that metabolic stress can correlate with the immune response. Neutrophil, lymphocyte, and thrombocyte counts increased in both athletes due to the BVb matches, reflecting a classic intensity-dependent reaction in WBCs first observed during the 1902 Boston Marathon [24,25]. Neutrophils and A1AG exhibited a slower recovery, while the short recovery sessions were nearly sufficient to restore lymphocytes and thrombocytes to the baseline levels. To our knowledge, this delayed recovery of neutrophils has been documented in males across various exercise intensities but has not yet been reported in female beach sports athletes [26]. Cell damage can induce and be induced by inflammation. Regarding A1AG, we observed an upregulation due to the BVb matches in female athletes, a finding that we previously showed in various indoor and outdoor sports [27,28].

The observed heightened inflammatory response was accompanied by increased systemic stress hormones, ACTH and cortisol, as observed in other sports [29,30]. In our study, ACTH and cortisol levels rose sharply, followed by a rapid decline during short recovery sessions. This swift increase and decrease after stress cessation may reflect the athletes’ effective stress recovery, as previously reported, as those who maintain higher fitness levels typically show shorter HPA (hypothalamic–pituitary–adrenal) activation in response to various stressors [31]. Thus, the observed HPA activation and subsequent interruption may provide the biochemical validation of the adaptation level of the analyzed world-class athletes to the stress induced by the BVb matches. Notably, the elevations of these hormones, particularly ACTH (up to 400%), were similar to a study with male triathletes undergoing a high-intensity cycle ergometer-exercise at 70% VO_2_max for 1 h, followed by an incremental increase of 25 W every 2 min until exhaustion [29]. In addition to correlating with exercise intensity and duration, measuring glucocorticoids in sports is essential due to their effects on the immune response and metabolism, particularly the glycemic response [30,32].

### 4.4. Glucose and Energy Metabolism

As expected, the acute rises in ACTH and cortisol were accompanied by a significant increase in glycemia. The BVb triggered an increase in glycemia, accompanied by a proportional decrease in insulin, demonstrating a classic glycemic response to exercise that has now been observed in female beach volleyball athletes. For a comparison between outdoor sports, we observed a glucose increase of 35% to 50% in this study, but we found previously that male canoeing athletes showed a higher rise (approximately 70%) with lower elevations in ACTH and cortisol [33]. It is important to note that although influenced by glucocorticoids, the complex glycemic response during exercise involves an interplay of inter-organ glucose output and uptake, primarily in the liver and skeletal muscles [34]. During intense exercise, the increased liver glucose output driven by glucocorticoids and sympathetic stimulation can exceed the systemic uptake, resulting in elevated glycemia and decreased insulinemia [35].

The high-intensity nature of a BVb competition day is evident in the measured metabolic and immune response. The increase in classical markers of exercise intensity, such as lactate and ammonium, corroborates this fact [36,37]. Lactate, a byproduct of the glycolytic pathway linked to a lower ATP/ADP ratio—a crucial signaling factor that modulates enzyme activity in the glycolytic pathway—also increased during the games due to exercise [38]. The BVb-induced increase occurred mainly in the second match, which can reinforce that the second match is more intense from the perspective of biochemical effects, possibly due to cumulative strain. A similar response was observed for ammonium, which increased more in the second match than in the first. However, it should be noted that in both athletes, the ammonium peak was found during the long recovery, displaying a delayed response compared to lactate. Ammonium can be produced through various biochemical pathways during exercise, notably including the deamination of amino acids and the degradation of AMP [39]. Classically, AMP degradation is predominantly observed during high-intensity exercises, which counteracts the elevated AMP concentrations arising from the increased myokinase activity prompted by a rapid decline in the intracellular ATP/AMP ratio [36]. As a terminal metabolite of AMP, urate serves as an indirect indicator of myokinase activity [10]. Notably, urate levels increased in both athletes during the second match, following the increase in ammonium levels. This observation suggests that AMP degradation significantly influenced ammonium production, indicating a component of high-intensity exercise associated with BVb. However, during the LRec, the substantial elevation in the ammonium concentration was not matched by a corresponding increase in urate, indicating that amino acid deamination also plays a critical role in energy metabolism on a BVb competition day.

### 4.5. Amino Acids Response

Alanine, glutamate, and glutamine are major blood amino acids essential in anaplerosis and ammonia metabolism. In our protocol, the matches prompted an elevation in alanine and glutamate levels, whereas the alterations in glutamine concentrations were not uniform among the athletes. This variability in the response among these amino acids is not uncommon, considering that although their blood concentrations may increase during acute exercise, they are also subject to depletion in prolonged exercise due to sustained muscle uptake and deamination reactions [40,41]. Notably, in this study, we observed significant changes in glutamate, while glutamine showed minor variation. It is important to highlight that glutamate has a blood concentration much lower than that of glutamine (~5%, as shown in Figure 7), making its concentrations more susceptible to fluctuations due to release or uptake by different organs. This contrasts with a previous holistic analysis involving multiple sports, where our group found that glutamate typically exhibits a lower responsiveness to acute exercise compared to alanine and glutamine [28]. Conversely, it was already observed that intracellular and blood concentrations of glutamine may diminish during exercise by deamination, leading to the formation of glutamate and ammonium, both of which increased in our investigation [42]. These discrepancies highlight the importance of evaluating the unique demands placed by each sport, growing the significance of more metabolic investigations in neglected sports, such as BVb.

The impact of the BVb protocol on blood amino acids extended to branched-chain amino acids (BCAAs, i.e., isoleucine, leucine, valine) and the aromatics (AAA, i.e., phenylalanine, tryptophan, tyrosine). Among the BCAAs, valine exhibited the most significant alterations, increasing up to 160% or 210%, depending on the athlete, during the protocol. Conversely, isoleucine and leucine displayed decreasing trends throughout the protocol. Notably, the pattern of BCAA response was consistent among the athletes. In one previous investigation, our group showed that total BCAA levels may not undergo substantial acute changes post-exercise across various sports [28].

The analysis of acute changes in BCAA is particularly relevant when considered alongside the response of AAA; this BCAA/AAA ratio is classically referred to as the Fischer ratio. The concentration ratio of these amino acids in the bloodstream influences their transport across the blood–brain barrier (BBB), allegedly affecting neurotransmitter synthesis [43]. A decrease in this ratio is associated with a higher likelihood or severity of mental fatigue development. Following the BVb matches, both athletes experienced a reduction in the Fischer ratio, with Athlete 1 and Athlete 2 exhibiting declines of 30% and 10%, respectively, from baseline to the first recovery stage (SRec1). Athlete 1 demonstrated a return to initial levels by the time of pre-match 2. The second match elicited a similar response in Athlete 1 compared to the first match; however, Athlete 2 displayed a more pronounced decline, with the Fischer ratio dropping by 35% to 40% during post-match 2 and Srec2.

Our findings indicated that serotonin acutely increased following the analyzed BVb matches. At the same time, blood tryptophan consistently decreased after these events (except for in Athlete 1 in the first match), suggesting an increased uptake of tryptophan into the central nervous system (CNS) from the bloodstream. As proposed by Newshome, another indirect marker of central fatigue that could be analyzed is tryptophan itself, which competes with BCAA for transport across the BBB [44]. Tryptophan serves as a precursor to serotonin, and it is well established that the acute increase in serotonin production during exercise plays a critical role in the onset of central fatigue, a phenomenon often referred to as the “serotonin hypothesis” [45]. Newsholme’s central fatigue hypothesis is controversial but accepted by many scientists. In our study, although we did not directly measure central serotonin production, we did assess blood levels of both serotonin and tryptophan. However, it is important to note that since we only measured them in the blood, we are dealing with a net balance of the uptake and release of tryptophan, and future studies with elite athletes should merge these measurements with psychometric or performance data.

### 4.6. The Same Protocol and Conditions but Different Responses: The Role of n-of-1 Monitoring in Understanding Athletes’ Real-World Performance and Metabolic Response

Personalized medicine has become a growing trend in scientific research. Transitioning from population studies to identifying predictive markers for various conditions that can categorize individuals into more homogeneous groups is an effective strategy for personalized interventions—the smaller and more specific the subgroup, the greater the potential effectiveness of the intervention. For example, this approach has developed new study models, such as n-of-1 trials. It has become increasingly evident in sports research that athletes following the same exercise protocol in identical environments can show vastly different physiological responses, especially when considering elite athletes [46]. This highlights the need to differentiate between internal and external loads comprising the total training load [47]. However, it is important to recognize that the limited sample size in this type of study may limit the external validity of these data to other athlete populations. Therefore, readers should consider that this study involves a highly specialized population, and the observed responses should be interpreted appropriately.

While the athletes’ selected biomarkers of injury and stress were comparable at rest, they diverged significantly during the matches (Figure 8). For example, a lower increase in ACTH in Athlete 2 led to a 60% higher cortisol response after the first match. During the second match, when the ACTH response was similar between the athletes, the cortisol response was three times higher for Athlete 2. This suggests that the athletes experienced different stress responses during the protocol, with Athlete 2 being more sensitive to ACTH signaling. Conversely, although Athlete 2 had a higher cortisol elevation after the second match, this increase lasted for a shorter time compared to Athlete 1. It is important to note that our discussion is primarily focused on metabolism, but assessing the exercise-induced increases in these stress hormones is intricate due to the known influence of psychological factors; for instance, individuals with anxiety tend to exhibit more pronounced elevations in hormone levels following exercise [48].

The responses between the two athletes after the first match were more similar than those observed after the second match, despite having similar nutrition and training routines, as well as undergoing a similar protocol. Some differences suggest that Athlete 2 experienced a more pronounced cumulative impact. For instance, lactate levels changed similarly after the first match, but Athlete 2 showed a 45% higher increase after the second match. Additionally, the evaluation of myoglobin, the most sensitive marker for muscle microinjury, did not differ significantly between the athletes after matches 1 and 2, as shown in Figure 8. However, it should be noted that the MB peak was delayed until the short recovery session, during which Athlete 2 exhibited a 40% higher elevation. Moreover, the protocol-induced increase in ammonium was matched with an increase in urate in Athlete 2 but not in Athlete 1, suggesting that the primary source of ammonium for Athlete 1 was amino acid deamination. At the same time, Athlete 2 displayed a greater role of the purine cycle.

In this sense, it is clear that even under comparable external conditions and exercise loads, players can display different responses to exercise. Therefore, coaches and sports staff should avoid standardizing training and recovery protocols for athletes, even when they share similar lifestyle habits and anthropometric characteristics and have participated in the same matches. For instance, athletes who demonstrate heightened hormonal stress responses, such as Athlete 2 in this study, may benefit from additional recovery days or alternative recovery modalities. Consequently, the training intensity, volume, and exercise type can be tailored based on real-time biomarker feedback. The same individualized approach should be applied to dietary interventions and electrolyte replenishment strategies. Indeed, we have observed that this phenomenon not only occurs during exercise but also in other models of hypermetabolic and inflammatory stress, such as liver failure, cancer, inflammatory arthritis, and sepsis, highlighting the importance of precision medicine.

## 5. Conclusions

To the best of our knowledge, this is a pioneering metabolic and immune investigation into in-field beach volleyball with world-class athletes, especially considering the lack of studies on any beach sports. As an exploratory study, the findings must be interpreted with caution, considering the ex post facto nature of the research and the highly specialized population examined. We have described and discussed the responses of clinically significant markers. Notably, both athletes exhibited a trend toward electrolyte disturbances, especially hypokalemia. Given that proper electrolyte balance is essential for optimizing performance and preventing health issues and that beach sports can pose a higher risk for these disturbances, our data can reinforce the need for BVb players to monitor these changes, particularly concerning potassium. Moreover, our data highlight that extended recovery periods may not fully restore athletes’ biological marker signatures, as demonstrated by the response of the muscle microinjury markers. Understanding the kinetics of these markers is essential for gaining insights into the physiological challenges faced by athletes and the environmental effects on their performance.

We also highlighted selected varying responses among the athletes. This discussion highlights the need for the individual and longitudinal monitoring of athletes, as in real-world scenarios, they can have different responses and are subject to multiple variables that are impossible to replicate in laboratory studies. Moreover, large studies and statistical analyses evaluating group responses can obscure unique responses, potentially misleading direct data translation. However, the limited sample size of this study constrains the direct generalizability of these findings to the broader population. Consequently, similarly to clinical practice, sports researchers and athletes’ support teams must recognize the generally expected responses from well-controlled studies while acknowledging that numerous real-world variables may limit their direct translation and applicability.

## 6. Limitations

As an ex post facto sportomics investigation conducted during a real training session for the Olympics, we did not control certain conditions, such as diet (the macronutrients are distribution described in the methods section) or sleep. In the discussion on central fatigue, we emphasize that concurrent psychometric assessments or measurements of central nervous system markers would be essential to complement the blood biochemical data and facilitate practical applications. The discussion surrounding tryptophan, serotonin, and the Fischer ratio aims to highlight the importance of these measurements in sports science and to describe their behavior in this highly specialized population, rather than to confirm any hypothesis regarding the onset of fatigue. Moreover, considering that the seven sample collections were conducted within a single day, it is important to acknowledge the potential influence of time on the hormone analysis. The environment also presents multiple factors that can affect an athlete’s response during the games, such as temperature, humidity, the sandy surface, and wind. Since it was not our intention to control these variables, the study is limited in discussing their individual effects, instead capturing the overall and cumulative impact of these factors. The study focused on only two athletes, both at the top level of the sport, representing a traditional beach volleyball nation. Therefore, the findings should be interpreted with caution. While they can help generate hypotheses for future studies or monitor athletes in real scenarios, the data should not be directly translated, especially for amateur athletes. Data translation must always consider the unique characteristics of each athlete, even when derived from laboratory-controlled studies, making this consideration even more crucial here. Consequently, the study’s results should not be used to discuss mechanisms or draw definitive conclusions but rather to observe fundamental differences in a non-controlled environment, raise new hypotheses for future investigation, reinforce previously published data, and highlight the need for more n-of-1 monitoring.

## 7. Future Perspectives

This exploratory study provides insight into the physiological responses of two female Olympic medalists in a sport that has been largely neglected in scientific research. The findings presented here warrant further investigation through various approaches. Studies involving other elite athletes in beach volleyball and related beach sports would be valuable for comparing responses across athletes at similar levels. In this context, n-of-1 studies or team evaluations could be beneficial for monitoring athletes during their actual training sessions and competitions. Additionally, we advocate for controlled studies to examine the influence of individual variables on athletes’ metabolic and inflammatory responses, not only in beach volleyball but also in other outdoor sports and models of beach sports. For instance, the potential trend toward hypokalemia observed in this study should be further explored concerning factors such as solar radiation, humidity, and the use of UV-protective clothing or sunscreen. We also recognize the importance of monitoring these athletes over a longer period, as several analytes related to metabolic stress did not recover within the analyzed timeframe.

## Figures and Tables

**Table 1:** A summary of the analyzed blood biomarkers.

Blood Biomarkers Analyzed	
Group	Biomarker
Red Sector	Erythrocytes, Hemoglobin, Hematocrit, Potassium, Magnesium, Chloride, Sodium.
Cell Damage	CK, MB, LDH, CK-MB, ALT, ALP, AST, GGT.
Inflammation	Neutrophils, Lymphocytes, A1AG, Thrombocytes.
Hormones	ACTH, Cortisol, Serotonin, Prolactin.
General Metabolism	Glucose, Insulin, Lactate, Ammonium, Urate, Urea, Creatinine.
Amino Acids	Glutamine + Histidine, Glutamate, Alanine, Leucine, Isoleucine, Valine, Tyrosine, Tryptophan, Phenylalanine.

**Table 2:** The urine specific gravity (USG) values over the course of the simulated day of competition demonstrated a trend of hypohydration throughout the protocol.

	Pre-Match 1	Post-Match 1	SRec 1	Pre-Match 2	Post-Match 2	SRec 2	LRec	Reference Range for Dehydrated
USG								
Athlete 1	1.022	1.009	1.007	1.024	1.019	1.024	1.024	1.021–1.030
Athlete 2	1.003	1.012	1.024	1.011	1.014	1.026	1.024	1.021–1.030

## Data Availability

The raw data supporting the conclusions of this article will be made available by the authors on request. The data are not publicly available due to privacy reasons.

## References

[1] Hank M., Cabell L., Zahalka F., Miřátský P., Cabrnoch B., Mala L., Maly T. (2024). Differences in external load among indoor and beach volleyball players during elite matches. PeerJ.

[2] Jenkins E.J., Campbell H.A., Lee J.K.W., Mündel T., Cotter J.D. (2023). Delineating the impacts of air temperature and humidity for endurance exercise. Exp. Physiol..

[3] Otani H., Kaya M., Tamaki A., Goto H., Maughan R.J. (2019). Exposure to high solar radiation reduces self-regulated exercise intensity in the heat outdoors. Physiol. Behav..

[4] Alkemade P., DE Korte J.Q., Bongers C.C.W.G., Daanen H.A.M., Hopman M.T.E., Janssen T.W.J., Eijsvogels T.M.H. (2023). Humid Heat Equally Impairs Maximal Exercise Performance in Elite Para-Athletes and Able-Bodied Athletes. Med. Sci. Sports Exerc..

[5] Bahr R., Reeser J.C., Volleyball F.I.d. (2003). Injuries among world-class professional beach volleyball players. The Fédération Internationale de Volleyball beach volleyball injury study. Am. J. Sports Med..

[6] Rallis E., Tertipi N., Sfyri E., Kefala V. (2024). Prevalence of Skin Injuries in Beach Volleyball Athletes in Greece. J. Clin. Med..

[7] Muniz-Santos R., França A., Jurisica I., Cameron L.C. (2023). From Microcosm to Macrocosm: The -Omics, Multiomics, and Sportomics Approaches in Exercise and Sports. OMICS.

[8] Sellami M., Elrayess M.A., Puce L., Bragazzi N.L. (2021). Molecular Big Data in Sports Sciences: State-of-Art and Future Prospects of OMICS-Based Sports Sciences. Front. Mol. Biosci..

[9] França T.C.L., Muniz-Santos R., Caetano L.C., Souza G.H.M.F., Goulart H.F., Assis M., Bottino A., Bassini A., Santana A.E.G., Prado E.S. (2023). A sportomics soccer investigation unveils an exercise-induced shift in tyrosine metabolism leading to hawkinsinuria. Front. Nutr..

[10] Gonçalves L.C.O., Magalhães-Neto A.M., Bassini A., Prado E.S., Muniz-Santos R., Verli M.V.A., Jurisica L., Lopes J.S.S., Jurisica I., Andrade C.M.B. (2022). Sportomics suggests that albuminuria is a sensitive biomarker of hydration in cross combat. Sci. Rep..

[11] Bongiovanni T., Pintus R., Dessì A., Noto A., Sardo S., Finco G., Corsello G., Fanos V. (2019). Sportomics: Metabolomics applied to sports. The new revolution?. Eur. Rev. Med. Pharmacol. Sci..

[12] Bragazzi N.L., Khoramipour K., Chaouachi A., Chamari K. (2020). Toward Sportomics: Shifting from Sport Genomics to Sport Postgenomics and Metabolomics Specialties. Promises, Challenges, and Future Perspectives. Int. J. Sports Physiol. Perform..

[13] Bassini A., Cameron L.C. (2014). Sportomics: Building a new concept in metabolic studies and exercise science. Biochem. Biophys. Res. Commun..

[14] Miller G.D., Teramoto M., Smeal S.J., Cushman D., Eichner D. (2019). Assessing serum albumin concentration following exercise-induced fluid shifts in the context of the athlete biological passport. Drug Test. Anal..

[15] Périard J.D., Eijsvogels T.M.H., Daanen H.A.M. (2021). Exercise under heat stress: Thermoregulation, hydration, performance implications, and mitigation strategies. Physiol. Rev..

[16] Resende N.M., de Magalhães Neto A.M., Bachini F., de Castro L.E., Bassini A., Cameron L.C. (2011). Metabolic changes during a field experiment in a world-class windsurfing athlete: A trial with multivariate analyses. Omics.

[17] Schuth G., Szigeti G., Dobreff G., Pašić A., Gabbett T., Szilas A., Pavlik G. (2024). Football Movement Profile-Based Creatine-Kinase Prediction Performs Similarly to Global Positioning System-Derived Machine Learning Models in National-Team Soccer Players. Int. J. Sports Physiol. Perform..

[18] Saita Y., Hattori K., Hokari A., Ohyama T., Inoue J., Nishimura T., Nemoto S., Aoyagi S. (2023). Plasma myoglobin indicates muscle damage associated with acceleration/deceleration during football. J. Sports Med. Phys. Fitness.

[19] Bassini-Cameron A., Sweet E., Bottino A., Bittar C., Veiga C., Cameron L.C. (2007). Effect of caffeine supplementation on haematological and biochemical variables in elite soccer players under physical stress conditions. Br. J. Sports Med..

[20] Tiller N.B., Stringer W.W. (2023). Exercise-induced increases in “liver function tests” in a healthy adult male: Is there a knowledge gap in primary care?. J. Family Med. Prim. Care.

[21] Thorpe R., Sunderland C. (2012). Muscle damage, endocrine, and immune marker response to a soccer match. J. Strength. Cond. Res..

[22] Petibois C., Cazorla G., Poortmans J.R., Déléris G. (2002). Biochemical aspects of overtraining in endurance sports: A review. Sports Med..

[23] Baird M.F., Graham S.M., Baker J.S., Bickerstaff G.F. (2012). Creatine-kinase- and exercise-related muscle damage implications for muscle performance and recovery. J. Nutr. Metab..

[24] Larrabee R.C. (1902). Leucocytosis after violent Exercise. J. Med. Res..

[25] Haller N., Behringer M., Reichel T., Wahl P., Simon P., Krüger K., Zimmer P., Stöggl T. (2023). Blood-Based Biomarkers for Managing Workload in Athletes: Considerations and Recommendations for Evidence-Based Use of Established Biomarkers. Sports Med..

[26] Neves P.R.D.S., Tenório T.R.D.S., Lins T.A., Muniz M.T.C., Pithon-Curi T.C., Botero J.P., Do Prado W.L. (2015). Acute effects of high- and low-intensity exercise bouts on leukocyte counts. J. Exerc. Sci. Fit..

[27] Anderson L., Razavi M., Pope M.E., Yip R., Cameron L.C., Bassini-Cameron A., Pearson T.W. (2020). Precision multiparameter tracking of inflammation on timescales of hours to years using serial dried blood spots. Bioanalysis.

[28] Muniz-Santos R., Bassini A., Falcão J., Prado E., Martin L., Chandran V., Jurisica I., Cameron L.C. (2024). Sportomics Analyses of the Exercise-Induced Impact on Amino Acid Metabolism and Acute-Phase Protein Kinetics in Female Olympic Athletes. Nutrients.

[29] Inder W.J., Hellemans J., Swanney M.P., Prickett T.C., Donald R.A. (1998). Prolonged exercise increases peripheral plasma ACTH, CRH, and AVP in male athletes. J. Appl. Physiol. (1985).

[30] Soslu R., Uysal A., Devrilmez M., Can Çuvalcıoğlu İ., Doğan A.A., Karaburgu S., Taş M. (2023). Effects of high-intensity interval training program on pituartry function in basketball players: A randomized controlled trial. Front. Physiol..

[31] Hare B.D., Beierle J.A., Toufexis D.J., Hammack S.E., Falls W.A. (2014). Exercise-associated changes in the corticosterone response to acute restraint stress: Evidence for increased adrenal sensitivity and reduced corticosterone response duration. Neuropsychopharmacology.

[32] Deuster P.A., Petrides J.S., Singh A., Lucci E.B., Chrousos G.P., Gold P.W. (1998). High intensity exercise promotes escape of adrenocorticotropin and cortisol from suppression by dexamethasone: Sexually dimorphic responses. J. Clin. Endocrinol. Metab..

[33] Coelho W.S., Viveiros de Castro L., Deane E., Magno-França A., Bassini A., Cameron L.C. (2016). Investigating the Cellular and Metabolic Responses of World-Class Canoeists Training: A Sportomics Approach. Nutrients.

[34] Hargreaves M., Spriet L.L. (2020). Skeletal muscle energy metabolism during exercise. Nat. Metab..

[35] Richter E.A., Sylow L., Hargreaves M. (2021). Interactions between insulin and exercise. Biochem. J..

[36] Wilkinson D.J., Smeeton N.J., Watt P.W. (2010). Ammonia metabolism, the brain and fatigue; revisiting the link. Prog. Neurobiol..

[37] Domínguez R., Maté-Muñoz J.L., Serra-Paya N., Garnacho-Castaño M.V. (2018). Lactate Threshold as a Measure of Aerobic Metabolism in Resistance Exercise. Int. J. Sports Med..

[38] Rabinowitz J.D., Enerbäck S. (2020). Lactate: The ugly duckling of energy metabolism. Nat. Metab..

[39] Holeček M. (2022). Muscle Amino Acid and Adenine Nucleotide Metabolism during Exercise and in Liver Cirrhosis: Speculations on How to Reduce the Harmful Effects of Ammonia. Metabolites.

[40] Berg A., Keul J. (1980). Serum Alanine During Long-Lasting Physical Exercise. Int. J. Sports Med..

[41] Castell L.M., Newsholme E.A. (1998). Glutamine and the effects of exhaustive exercise upon the immune response. Can. J. Physiol. Pharmacol..

[42] Mourtzakis M., Graham T.E. (2002). Glutamate ingestion and its effects at rest and during exercise in humans. J. Appl. Physiol. (1985).

[43] Fischer J.E., Rosen H.M., Ebeid A.M., James J.H., Keane J.M., Soeters P.B. (1976). The effect of normalization of plasma amino acids on hepatic encephalopathy in man. Surgery.

[44] Newsholme E.A., Blomstrand E. (2006). Branched-chain amino acids and central fatigue. J. Nutr..

[45] Cordeiro L.M.S., Rabelo P.C.R., Moraes M.M., Teixeira-Coelho F., Coimbra C.C., Wanner S.P., Soares D.D. (2017). Physical exercise-induced fatigue: The role of serotonergic and dopaminergic systems. Braz. J. Med. Biol. Res..

[46] Lima-Alves A., Claudino J.G., Boullosa D., Couto C.R., Teixeira-Coelho F., Pimenta E.M. (2022). The relationship between internal and external loads as a tool to monitor physical fitness status of team sport athletes: A systematic review. Biol. Sport.

[47] Impellizzeri F.M., Marcora S.M., Coutts A.J. (2019). Internal and External Training Load: 15 Years On. Int. J. Sports Physiol. Perform..

[48] Gerra G., Volpi R., Delsignore R., Caccavari R., Gaggiotti M.T., Montani G., Maninetti L., Chiodera P., Coiro V. (1992). ACTH and beta-endorphin responses to physical exercise in adolescent women tested for anxiety and frustration. Psychiatry Res..

