# Correction: Muniz-Santos et al. The Influence of Competition Day Loads on the Metabolic and Immune Response of Olympic Female Beach Volleyball Athletes: A Sportomics Analysis. *Nutrients* 2025, *17*, 1924

**DOI:** 10.3390/nu17182978

**Published:** 2025-09-17

**Authors:** Renan Muniz-Santos, Flavio Bachini, Adriana Bassini, P. C. B. Alexandre, Igor Jurisica, Vinod Chandran, L. C. Cameron

**Affiliations:** 1 Lorraine Protein Biochemistry Group, Graduate Program in Neurology, Gaffrée e Guinle University Hospital, Rio de Janeiro 20270-004, RJ, Brazil; renanmuniz@edu.unirio.br (R.M.-S.); pedro.alexandre@unirio.br (P.C.B.A.); 2 Laboratory of Protein Biochemistry, The Federal University of the State of Rio de Janeiro, Rio de Janeiro 20290-250, RJ, Brazil; f.bachini@gmail.com (F.B.); bassiniadriana@gmail.com (A.B.); 3 Osteoarthritis Research Program, Division of Orthopedic Surgery, Schroeder Arthritis Institute and Data Science Discovery Centre for Chronic Diseases, Krembil Research Institute, University Health Network, Toronto, ON M5T 0S8, Canada; juris@ai.utoronto.ca; 4 Departments of Medical Biophysics and Computer Science, Faculty of Dentistry, University of Toronto, Toronto, ON M5G IL7, Canada; 5 Institute of Neuroimmunology, Slovak Academy of Sciences, 845 10 Bratislava, Slovakia; 6 Gladman-Krembil Psoriatic Arthritis Program, Schroeder Arthritis Institute, Krembil Research Institute, University Health Network, Toronto, ON M5T 0S8, Canada; vinod.chandran@uhn.ca; 7 Division of Rheumatology, Department of Medicine, Institute of Medical Science, Department of Laboratory Medicine and Pathobiology, Faculty of Medicine, University of Toronto, Toronto, ON M5T 0S8, Canada

## Addition of an Author

Flavio Bachini was not included as an author in the original publication [1]. The corrected Author Contributions statement appears here:

**Author Contributions:** R.M.-S.: Visualization and Writing—Original Draft Preparation. F.B.: Investigation and Writing—Original Draft Preparation. A.B.: Investigation, Methodology, and Writing—Original Draft Preparation. P.C.B.A.: Supervision and Writing—Original Draft Preparation. I.J.: Conceptualization, Visualization, and Writing—Review and Editing. V.C.: Conceptualization and Writing—Review and Editing. L.C.C.: Conceptualization, Methodology, Supervision, Project Administration, Funding Acquisition, and Writing—Original Draft Preparation, Review, and Editing. All authors have read and agreed to the published version of the manuscript.

The authors state that the scientific conclusions are unaffected. The original publication has also been updated.
